# Automated detection of anterior cruciate ligament tears using a deep convolutional neural network

**DOI:** 10.1186/s12891-022-05524-1

**Published:** 2022-06-15

**Authors:** Yusuke Minamoto, Ryuichiro Akagi, Satoshi Maki, Yuki Shiko, Ryosuke Tozawa, Seiji Kimura, Satoshi Yamaguchi, Yohei Kawasaki, Seiji Ohtori, Takahisa Sasho

**Affiliations:** 1 Center for Preventive Medical Sciences, Chiba University, Chiba, Japan; 2 Department of Physical Therapy, Faculty of Health Science, Ryotokuji University, Urayasu, Japan; 3 Department of Orthopaedic Surgery, Graduate School of Medicine, Chiba University, 1-8-1 Inohana, Chuo-ku, Chiba, 260-8670 Japan; 4 Sportsmedics Center, Chiba University Hospital, Chiba, Japan; 5 Biostatistics Section, Clinical Research Center, Chiba University Hospital, Chiba, Japan; 6 Graduate School of Global and Transdisciplinary Studies, Chiba University, Chiba, Japan; 7 Faculty of Nursing, Japanese Red Cross College of Nursing, Tokyo, Japan

**Keywords:** Deep learning, Machine learning, Artificial intelligence, Anterior cruciate ligament, Magnetic resonance imaging

## Abstract

**Background:**

The development of computer-assisted technologies to diagnose anterior cruciate ligament (ACL) injury by analyzing knee magnetic resonance images (MRI) would be beneficial, and convolutional neural network (CNN)-based deep learning approaches may offer a solution. This study aimed to evaluate the accuracy of a CNN system in diagnosing ACL ruptures by a single slice from a knee MRI and to compare the results with that of experienced human readers.

**Methods:**

One hundred sagittal MR images from patients with and without ACL injuries, confirmed by arthroscopy, were cropped and used for the CNN training. The final decision by the CNN for intact or torn ACL was based on the probability of ACL tear on a single MRI slice. Twelve board-certified physicians reviewed the same images used by CNN.

**Results:**

The sensitivity, specificity, accuracy, positive predictive value and negative predictive value of the CNN classification was 91.0%, 86.0%, 88.5%, 87.0%, and 91.0%, respectively. The overall values of the physicians’ readings were similar, but the specificity was lower than the CNN classification for some of the physicians, thus resulting in lower accuracy for the human readers.

**Conclusions:**

The trained CNN automatically detected the ACL tears with acceptable accuracy comparable to that of human readers.

## Introduction

An anterior cruciate ligament (ACL) injury is one of the most common sports injuries [1, 2]. As with any other injuries, the diagnosis is based on the history and mechanism of the injury occurrence, followed by a physical examination [3]. A combination of several specific tests, such as the anterior drawer test, the Lachman test, and the pivot shift test, often provide sufficient information for the diagnosis [3]. Subsequent imaging tests are commonly performed for a definitive diagnosis, and magnetic resonance imaging (MRI) is the most reliable and least invasive modality [4]. Several studies report high accuracy in using MRI to diagnose cruciate ligament injuries and associated intra-articular pathologies [5, 6]. However, MRI interpretation in the knee joint is susceptible to variability among readers depending on the experience level, even when performed by a musculoskeletal radiologist or a sports orthopaedic surgeon. It has been reported that overall accuracy and specificity could improve with each year of additional training, thus suggesting that inexperienced physicians are at higher risk of misdiagnosis [7]. An accurate diagnosis of a torn ACL may be difficult for non-musculoskeletal radiologists, general orthopedic surgeons, or clinicians not specialized in the knee surgery field [8].

The development of computer-assisted image analysis technologies may offer a solution for diagnosing ACL rupture. Deep learning is a class of machine learning which has recently yielded breakthroughs in computer vision tasks. In particular, convolutional neural network (CNN)-based deep learning approaches are of interest in various areas, including medical imaging, and several applications support diagnostics created by CNN learning methods [9]. CNNs are designed to automatically and adaptively learn features from data through backpropagation using multiple building blocks, such as convolution layers, pooling layers, and fully connected layers. Due to large datasets’ availability and increased computing power, CNNs have outperformed conventional image analysis methods and resulted in significant progress in the medical imaging field. Recently, many clinical applications for CNNs have been reported in radiology for detection, classification, and segmentation tasks. However, the number of studies applying a CNN to knee MRIs is limited [10–12].

The development of technologies that can assist physicians in diagnosing ACL injury from a knee MRI would be beneficial. It would aid non-specialists who are not as familiar with knee injuries and could also support knee joint specialists in the diagnostic process. To implement these technologies in clinical practice, the diagnostic performance of the CNN must be reliable. At the same time, a versatile system that would work on a simple platform and with few requirements regarding the number and quality of the images would be desired.

This study aims to evaluate the accuracy of a CNN for the diagnosis of ACL ruptures using a single slice from a knee MRI. Furthermore, we compared the accuracy to that of experienced human readers. We hypothesized that the CNN could generate an accurate classification and thereby demonstrate the utility of this system to assist with ACL injury diagnosis.

## Methods

### Subject

The institutional review board has approved this research of the authors’ affiliated institutions. The need for consent from each patient was waived due to the study design as a retrospective analysis of anonymized imaging data. All patients who received arthroscopic surgery in our institution between June 2009 and October 2019 were eligible for the study. We included the patients with and without ACL injury, in which the diagnosis was confirmed by arthroscopy. Patients who did not receive preoperative imaging by either a 1.5 T (T) or a 3.0 T scanner were excluded. The patients with intact ACLs underwent arthroscopic surgery for other reasons, such as a discoid meniscus or meniscus tears. MR images of ACL tears were collected regardless of a complete or incomplete tear. The images of consecutive patients were retrospectively reviewed and included until both groups consisted of 100 images. The images were anonymized and extracted from the Picture Archiving and Communication System (PACS) for analysis.

### MRI dataset

The sagittal images of proton density-weighted MRIs were used for the CNN training. The images were extracted from Digital Imaging and Communications in Medicine (DICOM) files, acquired on either a 1.5 T or 3.0 T MRI scanner. Since the images were collected from multiple institutions, there was variation in the imaging protocol. The proton density-weighted MR images were obtained with the following parameters: repetition time (TR) = 2000–2400 ms; echo time (TE) = 20–30 ms; field of view (FOV) = 135–140 mm; matrix size = 192 × 192–416 × 416; slice thickness = 0.7–3.0 mm; slice gap = 0–0.3 mm.

### Image preprocessing for the CNN

**Fig. 1:**
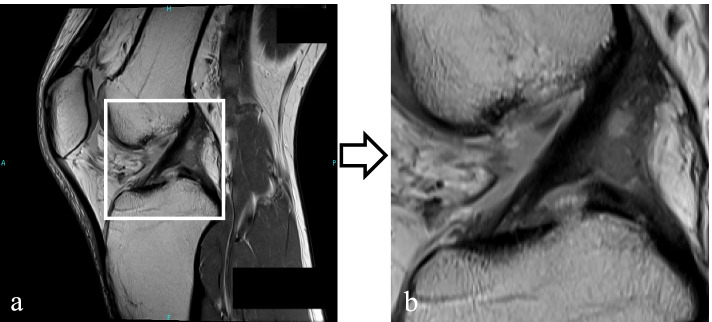
Image preparation. **a** The anterior border of the image was cropped at the articular capsule attachment of the anterior border of the tibia, and the posterior border was cropped at the tibial attachment of the posterior cruciate ligament. **b** The cropped image is used for reading

The MRIs were converted to JPEG format from the DICOM files. A single image slice was selected from each MRI series where the ACL was depicted continuously from the femoral attachment to the tibial attachment. According to the margin defined, the selected images were then cropped into the area of interest (Fig. 1). The anterior border was defined as the anterior end of the capsule attachment to the tibia, thus ensuring the tibial attachment of the ACL would be included. The posterior margin was defined at the posterior edge of the tibial attachment of the posterior cruciate ligament, ensuring the identification of the femoral attachment of the ACL. The upper and lower margins were defined to make the cropped area square with all sides of the same length and include both the femoral and tibial attachments in the area.

### CNN model

The CNN was built using Python programming language version 3.6.7 and Keras, version 2.2.4 with Google’s open-source deep learning framework Tensorflow, version 1.12.0 at the backend. In this study, the fine-tuning was performed using Xception, which had been trained on ImageNet images [13]. The weights of the first 108 layers were frozen, and the remaining layers were retrained using our dataset. The network was trained to 100 epochs with a learning rate of 0.1, but this was reduced if no improvement was seen. The model training convergence was monitored using cross-entropy loss. To increase the size of the dataset, we performed data augmentation. Using the ImageDataGenerator (https://keras.io/preprocessing/image/), all images were augmented with a random rotation between -20 and 20 degrees, a width and height shift range of 0.2 each, and a random horizontal flip. The CNN was trained on a computer equipped with a GeForce GTX 1650 Ti graphics processing unit (NVIDIA, Santa Clara, CA), a Core i5-3470 CPU 3.2 GHz (Intel, Santa Clara, CA), and 8 GB of random access memory.

### Performance evaluation

The performance of the CNN was evaluated with five-fold cross-validation. First, images of ACL tears were randomly divided into five equal-sized independent subgroups. In each iteration, four subgroups were designated as training data, and the remaining independent subgroup served as validation data. In the validation phase, the diagnostic performance to discriminate an ACL tear was assessed using the remaining independent subgroup. This cross-validation process was repeated five times.

### Classification

The final decision by the CNN was based on the probability of an ACL tear from a single MRI slice using the optimal cut-off point of the probability score.

### Image assessment by knee surgeons and radiologists

Ten board-certified knee surgeons (K1—K10 with 8 to 31 years of experience) and two board-certified radiologists (R1 and R2, with 16 and 14 years of experience, respectively) reviewed the same images used for training of the CNN. The evaluators were selected by including all board-certified knee surgeons in the institution and the two radiologists who routinely read musculoskeletal images. To directly compare the diagnostic ability between the CNN and human doctors under the same conditions, the readers were required to judge if the ACL was torn or intact from the single cropped slice image without any other information on patient history and results from the physical exams. Each reader assessed 200 images in a randomized order and labeled each image as 0 for intact and 1 for torn ACL.

### Statistics and data analysis

All statistical analyses were conducted using SAS (version 9.4 for Windows) and R (3.6.1). Based on the predictions, we calculated the true-positive, true-negative, false-positive, and false-negative rates. To evaluate the performance of the CNN, we plotted the receiver operating characteristic (ROC) curve and calculated the area under the curve (AUC). Then we calculated the sensitivity, specificity, and accuracy of the CNN and each knee surgeon and radiologist. The sensitivity, specificity, and accuracy were determined from the optimal threshold using the highest Youden index (sensitivity + specificity – 1) on the ROC analysis. Finally, the sensitivity, specificity, and accuracy of the diagnostic performance of the CNN, the knee surgeons, and the radiologists were compared using a McNemar test.

## Results

One hundred ninety-three patients were included in the study. One hundred MR images from 93 consecutive patients with an ACL injury (mean age 27.2 ± 10.6 years, 46 images in 45 males and 54 images in 48 females) and 100 MR images from 100 consecutive patients with an intact ACL (mean age 26.1 ± 11.9 years, 67 images in 67 males and 33 images in 33 females) were obtained (Table 1). Seven patients in the ACL injured group had two MRI scans in the presurgical period mainly due to a delay between the time of surgery and the initial injury. The clinical diagnoses before surgery in the intact ACL group are shown in Table 2.

**Table 1:** Patient characteristics

	Torn ACL group	Intact ACL group
n (patients)	93	100
Age	27.2 ± 10.6	26.1 ± 11.9
Sex (M/F)	45 / 48	67 / 33

**Table 2:** Clinical diagnoses before surgery in the intact ACL group

Meniscus tear	32
Tumor	12
Osteochondritis dissecans	6
Cartilage injury	8
Recurrent patella dislocation	3
Other	3

**Fig. 2:**
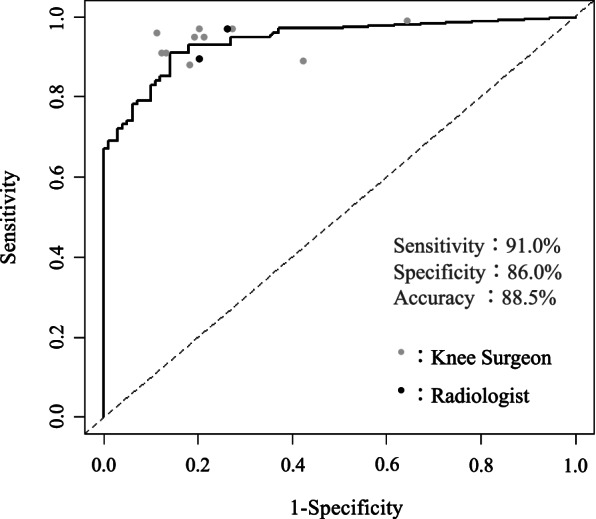
The ROC curve based on the CNN and physicians’ performance. AUC = 0.942 (95% CI, 0.911–0.973). ROC: receiver operating characteristic. CNN: convolutional neural network. AUC: area under the curve

The sensitivity, specificity, accuracy, positive predictive value, and the negative predictive value calculated from the interpretation results of the CNN as well as the knee surgeons and radiologists are shown in Table 3. The sensitivity, specificity, accuracy, positive predictive value and negative predictive value of the CNN reading was 91.0%, 86.0%, 88.5%, 87.0%, and 91.0%, respectively. The physicians’ overall values were similarly good, but the specificity was lower than the CNN reading for some physicians (K5 and K9), resulting in lower accuracy. The ROC curve created from the results of the CNN is presented in Fig. 2, and the physicians’ results are plotted. The AUC was 0.942 (95% confidence interval (CI), 0.911–0.973), and the cut-off of the probability score to detect a torn ACL by the CNN with the highest accuracy was 0.78.

**Table 3:** Sensitivity, specificity, accuracy, PPV, and NPV of the CNN and physicians

Evaluator (years of experience)	Sensitivity(%)	Specificity(%)	Accuracy(%)	PPV(%)	NPV(%)
CNN	91.0	86.0	88.5	87.0	91.0
K1 (31)	91.0	87.0	89.0	87.5	90.6
K2 (21)	96.0	89.0	92.5	89.7	95.7
K3 (17)	97.0	80.0	88.5	82.9	96.4
K4 (11)	95.0	79.0	87.0	81.9	94.0
K5 (9)	99.0	36.0	67.5	60.7	97.3
K6 (10)	91.0	88.0	89.5	88.3	90.7
K7 (9)	97.0	73.0	85.0	78.2	96.1
K8 (9)	95.0	81.0	88.0	83.3	94.2
K9 (9)	89.0	58.0	73.5	67.9	84.1
K10 (8)	88.0	82.0	85.0	83.0	87.2
R1 (16)	89.5	80.0	84.8	81.7	87.9
R2 (14)	97.0	74.0	85.5	78.9	96.1

*K* Knee surgeon, *R* Radiologist

The accuracy was compared between the CNN and each physician (Table 4). The accuracies obtained by two knee surgeons (K5 and K9) were below 80% and were significantly lower than the accuracy of the CNN.

**Table 4:** Difference in the Accuracy between the CNN and Physicians (McNemar test)

	Accuracy(%)	*P*-value
CNN	88.5	-
K1	89.0	0.8788
K2	92.5	0.1824
K3	88.5	1.0000
K4	87.0	0.6617
K5	67.5	< .0001*
K6	89.5	0.7456
K7	85.0	0.3173
K8	88.0	0.8759
K9	73.5	0.0001*
K10	85.0	0.2858
R1	84.8	0.6717
R2	85.5	0.3657

*K* Knee surgeon, *R* Radiologist

*p < 0.05

## Discussion

This study was conducted to evaluate the ability of the CNN to assist with the diagnosis of a torn ACL from a single cropped MRI image of the knee, using the results from an arthroscopic examination as the gold standard. Our system revealed sufficient capability to define the presence of ACL tears with better specificity compared to experienced human readers.

In a recent report on CNN-based MRI reading of ACL injuries, Bien et al. [14] tested a CNN model predicting an ACL tear from three slices of an MRI and reported a sensitivity of 75.9%, a specificity of 96.8%, and an accuracy of 86.7%. The reported AUC in this study was 0.965. More recently, Chang et al. [8] reported a sensitivity of 100%, a specificity of 93.3%, and an accuracy of 88.5% for a CNN model, which used five slices per MRI to define an ACL rupture. Our CNN model presented slightly inferior results in specificity but with a similar accuracy compared to these two reports. The difference between our study and these previous reports is that they used multiple MRI slices to predict the presence of an ACL tear. Considering the model’s utility in clinical practice, ease of implementation would be essential. The implementation would be affected by the number of images needed, the image preparation process, and the complexity of the training protocol. Our CNN model presented a similar ability to classify an ACL rupture from a single slice and required fewer training sessions. Thus the proposed method may be more easily implemented in clinical practice and would be expected to reduce errors, leading to more effective medical care. This would most apply to general orthopedic surgeons, trainees, and clinicians who are not in the field of knee surgery that may have poor diagnostic accuracy when reading the images.

Previous reports of MRI reading of ACL injuries by human readers have shown that the accuracy of orthopedic surgeons is 80 ~ 90%, and that of musculoskeletal radiologists is 92 ~ 98% [14–19]. In addition, it has been reported that the accuracy of the reading is not influenced by the magnetic field strength (either 1.5 T or 3.0 T) or the acquisition conditions of the MRI scanner [15, 16]. Compared to previous reports, the knee surgeon’s overall reading sensitivity in this study was good, with similar results in terms of specificity and accuracy. The results obtained by the two radiologists in our study were also comparable to previous reports.

By plotting the results from the knee surgeons and radiologists together with the ROC curve derived from the results of the CNN system, the overall performances of the human readers and the CNN were comparable. The McNemar test revealed a significant difference between the CNN and knee surgeons, indicating a lower specificity and inferior performance by two of the surgeons. The specificity obtained from the diagnosis of these two readers was below 80%, which may be insufficient in a clinical setting. However, it would be unfair to conclude that CNN was superior to human readers since the human readers were required to assess the images under unusually stringent conditions. Still, considering that the overall quality of the readers included in our study was reasonable compared to previous reports, we conclude that our CNN model would help screen ACL tears from a single MRI slice and with good sensitivity and specificity.

This study is not without limitations. First, we included only one MRI slice per exam and cropped the image to a small area, including the ACL. By this image modification, many MRI features of a torn ACL, such as tortuosity, bulging, bone bruise, and PCL bowing [20], were excluded from the image and were not taken into account. Since the CNN had limited information for training, the diagnostic ability may have been underestimated. Also, the additional process of cropping the image, instead of simply using the entire image sets, could be cumbersome in practice. A system to diagnose a torn ACL with an accuracy better than human readers, and a simplification of the training conditions with less image modification, would be ideal. Still, we consider it a strength of our system that an acceptable level of diagnostic assistance can be provided from the limited information of a single slice. Furthermore, the knee surgeons and radiologists who evaluated the images were required to make a judgment with less information than usual, which would have affected their diagnostic performance. The readers included in the current study determined the ACL tears with comparable quality to previous reports. Therefore, we consider that this limitation did not result in an overestimation of the diagnostic capability of the CNN. Finally, the number of images included in the study was relatively small. A larger number of patients would improve the quality and reliability. Still, by conducting the five-fold cross-validation and data augmentation, we were able to achieve an acceptable quality of MRI reading by our CNN model. From a future perspective, improved diagnostic performance would be expected by implementing more patients and incorporating clinical information.

## Conclusion

We developed a CNN system to diagnose ACL tears with acceptable accuracy, comparable to that of human readers. An artificial intelligence-based diagnostic model for MRI could help non-experts diagnose and determine if consultation with experts is needed for a suspected ACL injury. Further studies to automatically prepare the image for analysis and compare the single slice evaluation to multiple slice evaluation would be expected to improve the clinical application.

## Data Availability

The datasets used and analysed during the current study available from the corresponding author on reasonable request.

## Abbreviations

ACL: Anterior cruciate ligament
MRI: Magnetic resonance imaging
CNN: Convolutional neural network
DICOM: Digital Imaging and Communications in Medicine
TR: Repetition time
TE: Echo time
FOV: Field of view
ROC: Receiver operating characteristic
AUC: Area under the curve
